# Chemical proteomics, an integrated research engine for exploring drug-target-phenotype interactions

**DOI:** 10.1186/s12953-017-0129-x

**Published:** 2018-01-09

**Authors:** Ho Jeong Kwon, Peter Karuso

**Affiliations:** 1 Department of Biotechnology, Yonsei University, 50 Yonsei-ro, Seodaemun-gu, Seoul, 120-749 South Korea; 2 Department of Chemistry and Biomolecular Sciences, Macquarie University, Sydney, NSW 2109 Australia

Chemical proteomics has emerged as an integrated research engine for the discovery of new drugs and the agnostic identification of drug targets. It involves the development and combination of chemical tools, bioorthogonal techniques, phenotypic screens, target identification and validation, mode of action and chemoinformatics that together provide robust and high-throughput workflows for exploring drug-target-phenotype relationships. In particular, the focus is on the application of small molecules in proteomics and genome-wide techniques for the rapid identification of drug targets.

The special issue of Proteome Science highlights the state-of-the-art for workflows and applications of chemical proteomics with 3 review articles and 2 original research papers contributed by the leading scientists in the field.

Savistski and co-workers summarize the recent developments in thermal proteome profiling (TPP) [1]. This technique relies on the thermal stabilization of a protein when it is bound to its ligand and the detection of this stabilization through changes in that protein’s concentration compared to an identical experiment with no added drug. In this respect it is similar to the drug affinity responsive target stability (DARTS) assay, which measures the difference in protein hydrolysis by a non-specific protease with varying concentrations of a ligand [2]. Both assays can be done in live cells, in which case TPP is called the cellular thermal shift assay (CETSA) [3]. Combining CETSA with quantitative proteomics (using hypobaric tagging of the tryptic digest), provides a powerful platform for the identification of drug targets without the need for labeling of the ligand. Such techniques are at the vanguard of label-free techniques for the identification and validation of drug targets [4]. The review described variants of the general workflow with a focus on elucidation of drug-target interactions as a means to generate target hypotheses for hits from phenotypic screens. Because quantitative proteomics techniques have extended the scope of target identification to the whole proteome level, this approach is receiving attention as a strategy to discover new targets and an important method for cellular metabolic mapping. In this regard, TPP could provide a unique approach to unveiling thousands of cellular dynamic environments for drugs of interest.

Protein-protein and drug-protein interactions have been studied by a variety of techniques including affinity chromatography, peptide arrays, activity and affinity-based probes, drug Western and photoaffinity labeling [5]. In the next paper, Lee and co-workers comprehensively review recent progress in photoaffinity labeling (PAL) technologies, developed by Westheimer in 1962 [6], based on three different photocrosslinkers (PLs); diazirines, benzophenones and arylazides. In this outstanding review, research examples of each PL type are nicely provided against multiple different proteins of interests. This review also provides some important examples of quantitative proteomics in the study protein-protein interactions (PPIs) including general principles and future perspectives. Given the important role of PAL in studying PPIs, this review is timely for the readers in chemical proteomics fields, who should be thinking about this technique for the study of drug-protein and natural product-protein interactions.

In the third review, Kim and co-workers provide a targeted review on the use of the tetrazine ligation reactions in chemical proteomics. Since Sharpless introduced the idea of bioorthogonal “click chemistry” in 2001 [7], there has been over 20 different such reactions identified so far. This article reviews one of those bio-orthogonal cycloaddition reactions; the [4 + 2] inverse demand Diels-Alder cycloaddition (tetrazine ligation), highlighting the chemical features of this reaction in biology and summarizing the applications of tetrazine ligation for protein imagining and drug-target identification. The authors review the range of fluorophores available with tetrazine groups and their use in protein and small molecule labeling and compare this to Cu(I) and copper-free Huisgen ligations and the use of cleavable and photoaffinity-based linkers in probe design.

In addition to these three review articles, Thomas et al. examines the influence of specificity of bivalent kinase probes that contain either a SH2 or SH3 binder plus an ATP mimic. In this excellent example of the design, synthesis and application of dual functional chemical proteomics probes, the authors confirmed the affinity and selectivity of their bivalent inhibitors against Abl along with other off targets. They found bivalent inhibitor A-2 has improved affinity as well as selectivity over the original ATP competitive inhibitor, KAM. These results provide interesting molecular design strategy applicable to many other bivalent inhibitors/probes that could find utility in chemical proteomics. Finally, Karuso and Kwon presented their findings on the unbiased, genome wide identification of a human protein target for the new antibiotic Daptomycin (DAP). They used a phage display cDNA expression platform (reverse chemical proteomics) to screen selected human cancer cDNA libraries for human binding partners for DAP [8]. Using this approach they identified the ribosomal protein S19 (RPS19), to be most the most common binding partner for DAP in all the cell lines. They validated this finding with a number of in vitro experiments, to confirm a role for DAP-RPS19 interaction as a possible target for cancer therapy. This paper provides a nice case study of the utility of reverse chemical proteomics to explore new targets of drugs with biological relevance that is applicable for the iterative identification of new targets where their mechanisms or target(s) and completely unknown. These sorts of methods will become increasing important as the need to identify a preclinical drug’s targets becomes increasing important for drug optimization, mode of action and ultimately FDA approval.

Collectively, the papers of this special issue devoted to chemical proteomics nicely highlights and exemplifies the applications of this emerging research field that promises to overcome the hurdle to identify protein targets of biologically active small molecules with unknown modes of action. Chemical proteomics is also applicable to the functional study of proteomes, understanding modes of action and for the development of novel drugs modulating novel targets. We hope you will enjoy this special issue and trust that these articles will stimulate further developments in the field.
